# Gas gangrene and osteomyelitis of the foot in a diabetic patient treated with tea tree oil

**DOI:** 10.1186/1865-1380-4-14

**Published:** 2011-04-14

**Authors:** Derek R Cooney, Norma L Cooney

**Affiliations:** 1 Department of Emergency Medicine, Division of Hyperbaric Medicine, SUNY Upstate Medical University, EMSTAT Center/550 East Genesee, Syracuse, New York 13202, USA

## Abstract

Diabetic foot wounds represent a class of chronic non-healing wounds that can lead to the development of soft tissue infections and osteomyelitis. We reviewed the case of a 44-year-old female with a diabetic foot wound who developed gas gangrene while treating her wound with tea tree oil, a naturally derived antibiotic agent. This case report includes images that represent clinical examination and x-ray findings of a patient who required broad-spectrum antibiotics and emergent surgical consultation. Emergency Department (ED) detection of these complications may prevent loss of life or limb in these patients.

## Background

The lifetime incidence of diabetic foot ulcers may be as high as 25% [1]; however, gas gangrene is not common in these patients. The most common causative organism in gas gangrene is *Clostridium perfringens *[2]. It is also important to rule out underlying osteomyelitis. In patients with diabetic foot ulcers, *Streptococcus *group A, *Staphlococcus aureus *and *Pseudomonas *may be present. If surgical debridement and antibiotics are not effective, amputation may be required.

Tea tree oil, the essential oil of *Melaleuca alternifolia*, is a commonly available, naturally derived, topical antiseptic. Tea tree oil is known to possess antibiotic activity toward a broad spectrum of pathogens, including methicillin-resistant *Staphylococcus aureus *(MRSA) and *Candida albicans *[3].

## Case Presentation

A 44-year-old female with a history of diabetes presented to the Emergency Department complaining of increased right foot pain for 3-4 days with redness and swelling. She had been applying tea tree oil to the wound. Her vital signs were blood pressure: 91/50, heart rate: 111, respiratory rate: 20, temperature: 36.4°C and oxygen saturation: 100% on room air. There were swelling and inflammation to the right foot and a foul-smelling odor. Dark blisters were noted with erythema tracking up the lateral aspect of the leg. There was a 4 × 3 × 2.5 cm ulcer plantar surface of the foot with maceration of the periwound skin and a serosanguinous drainage that had a mild odor. She was able to plantar and dorsiflex, and had diminished fine sensation. The patient had 1+ dorsalis pedis pulses and normal capillary refill. Laboratory studies showed a WBC of 14.7, neutrophils 74% with 17% bands. Radiographs of the right foot showed subcutaneous and deep fascial emphysema of the foot with extension along the plantar surface. Irregularities consistent with osteomyelitis of the distal first metatarsal and distal second proximal phalanx were noted.

After admission, the patient was treated with clindamycin and underwent a transmetatarsal amputation. Blood cultures were negative, and wound cultures were deemed inconclusive. Wound infection developed, and the patient was treated with vancomycin and moxifloxacin. An ankle disarticulation was performed. The wound did well at that point and the rest of the hospital course, and outpatient management was unremarkable.

## Discussion

Diabetic foot ulcers are a significant complication and are credited with causing 85% of limb amputations among diabetics. In a review by Sing et al., limb amputation was associated with 39-80% 5-year mortality [1]. Diabetic foot ulcers are usually the result of some minor trauma that may be secondary to the patient's decreased sensation. Ulceration in areas of increased pressure is also common. Usually offloading, debridement, advanced wound care dressings and close follow-up result in improvement of these wounds. Hyperbaric oxygen therapy is also commonly used as an adjunct in the care of these wounds. These are chronic wounds and require weeks of therapy and numerous clinic visits. When care is not taken to prevent infection, the wounds can become deep, and osteomyelitis and serious soft tissue infection may occur.

### Tea Tree Oil

A particularly interesting element of this case is the patient's use of the home remedy, tea tree oil. This essential oil is bactericidal and known to have some broad-spectrum antibiotic affects [3-8]. Tea tree oil is one of a number of essential oils that possesses an active monoterpene constituent. In a study by Cox et al., the monoterpene in tea tree oil was shown to damage cell membranes and inhibit cellular respiration in *Escheria coli*, *Staphylococcus aureus *and *Candida albicans *[4]. It has also been shown to have activity against *Pseudomonas *species [6]. Tea tree oil has been demonstrated to have antibiotic effects on a number of bacteria, including important skin flora like methicillin-resistant *Staphylococcus aureus *[3]. In addition to its antiseptic, antibiotic and antifungal activity, tea tree oil also has some anti-inflammatory effects [7]. The antiseptic property of tea tree oil likely explains the lack of useful wound culture results in this case.

### Assessement

**Figure 1:**
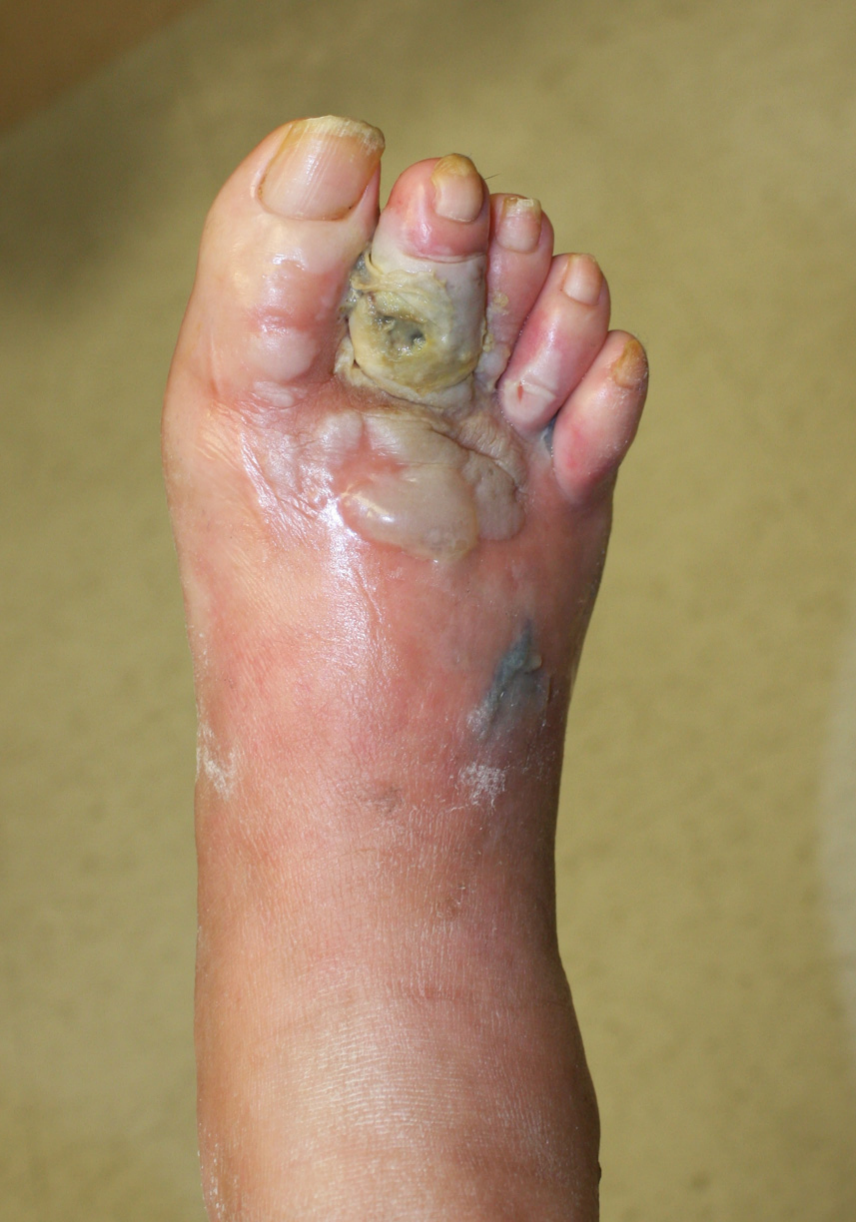
**Dorsal aspect reveals ulceration of secong digit, blisters and discoloration**.

**Figure 2:**
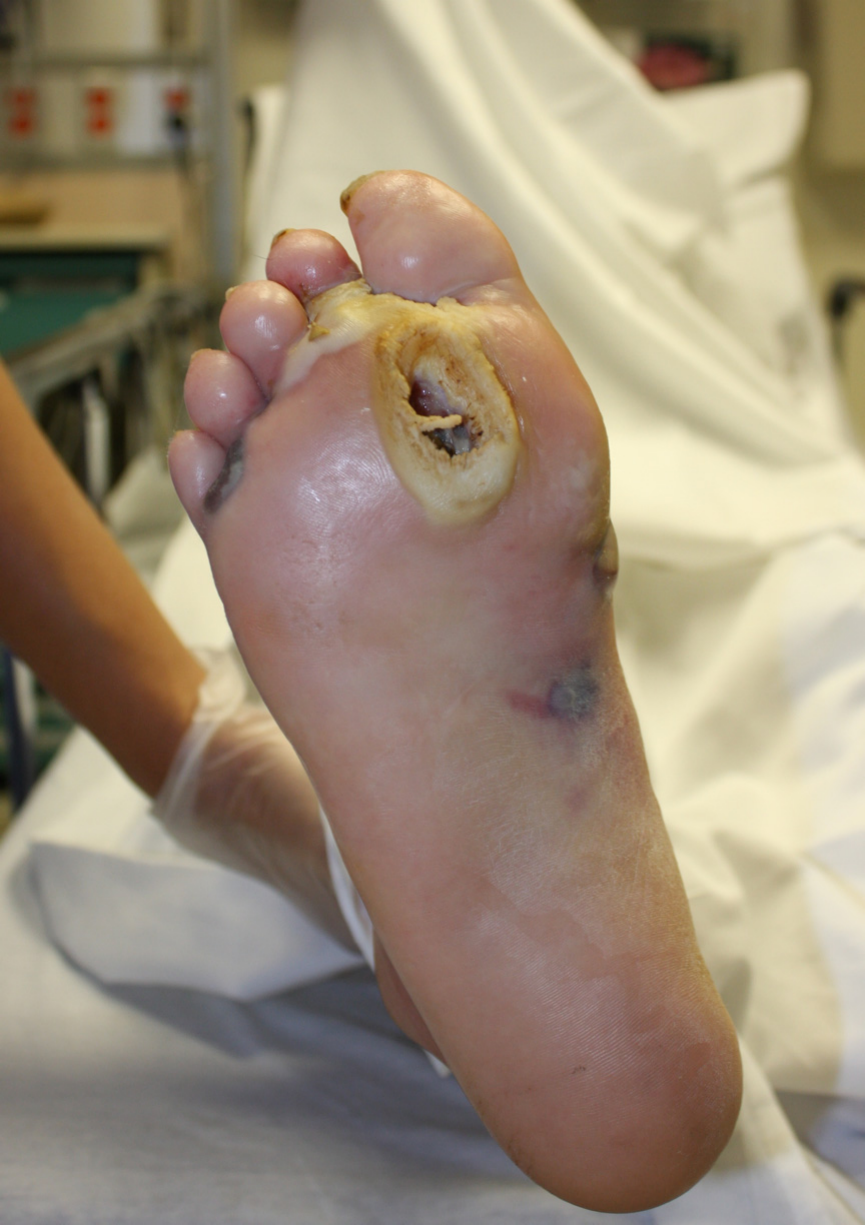
**Plantar aspect reveals diabetic foot ulcer**.

Pain is a common presenting complaint and may be the first sign in patients with gas gangrene. Bullae and the bluish skin discoloration are classic findings of gas gangrene and were noted have begun to show at the time of presentation of this case (Figures 1 and 2). Edema and crepitus are usually present at the time of diagnosis; however, some references state that as many as 50% of cases may not have discernable crepitus or gas on radiographs on initial presentation [9]. The patient's periwound areas were also quite macerated. This was due to the moderate amount of serosanguinous drainage the patient was having, which is also common with gas gangrene. This drainage is often described as having a "sickly sweet" odor.

**Figure 3:**
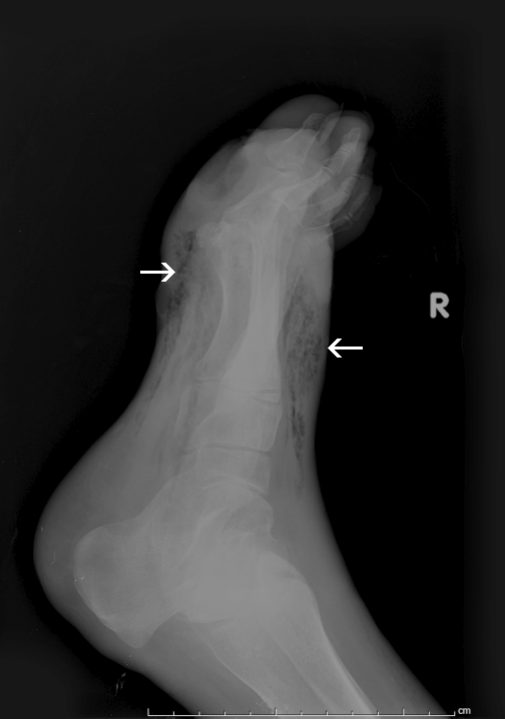
**X-ray reveals soft-tissue gas consistent with gangrene**.

X-ray images should be obtained in patients with diabetic foot ulcers to evaluate for the presence of osteomyelitis and gas in the soft tissues. Osteomyelitis was noted in this case; however, the soft tissue gas is much more prominent (Figure 3). The presence of gas on x-ray of the affected area should prompt the clinician to obtain images up to the next proximal joint in order to ascertain the extent of the infection [10]. In cases where gas is not seen, but deep space soft tissue infection is suspected, computed tomography (CT) or magnetic resonance imaging (MRI) may be appropriate.

### Management

Emergency department treatment for patients with signs of cellulitis with or without crepitus includes intravenous (IV) antibiotics. Antibiotic choice is varied and may be institutionally dependent. Broad-spectrum penicillins, such as *Piperacillin-tazobactam*, are most commonly employed [11]. Superficial wounds can be debrided, and eschers and fibrous caps removed, if the ED practitioner is skilled in these procedures. Drainage and debrided material should be cultured. Clinical response to therapy and culture results are usually used to direct changes in antibiotic therapy during the inpatient phase of management. It is important to remember that (IV) and oral antibiotics do not penetrate devascularized tissues. Gangrene and deep space infections require surgical debridement in the operating room. Some patient presentations may be complicated by sepsis or shock, and IV fluid therapy is indicated along with other supportive measures.

Aggressive debridement may be necessary for wounds associated with crepitus. Surgical exploration may help determine whether the condition is crepitant cellulitis verses gas gangrene. Necrotic and infected tissues, including muscle and fascia, should be removed, and healthy tissues should be preserved if possible. During surgical exploration, it may become apparent that amputation is necessary, which is the case in 25% of severe diabetic foot infections [10].

In addition to its role in chronic management of diabetic foot ulcers, hyperbaric oxygen therapy (HBOT) may have a role in the acute management of patients that develop infectious complications of their wound(s). A review by Kaide et al. states that HBOT has been shown to suppress alpha toxin of *Clostridium*, enhance leukocyte-killing activity, enhance destruction of anaerobic bacteria, suppress bacterial growth, enhance antibiotic effects, and improve tissue repair in poorly vascularized tissues [9]. The review also states that HBOT, when added to antibiotics and surgery, has also been found to reduce the rate of mortality and morbidity (including amputation). During surgery, patients undergoing HBOT were found to have clearer demarcation between viable and necrotic tissues, allowing for improved surgical debridement.

## Conclusion

Although an uncommon complication of diabetic foot ulcer, gas gangrene may develop in patients with these chronic non-healing wounds. Special care must be taken in the ED evaluation of these wounds to rule out the diagnosis of soft tissue infections, as well as osteomyelitis. The management of gas gangrene requires rapid recognition and immediate therapy. In addition to broad spectrum antibiotics and surgical consultation, the ED physician may also consider consulting for HBOT and ICU evaluations if appropriate.

## Competing interests

The authors declare that they have no competing interests.

## Authors' contributions

NC participated in the care of the patient and provided case details. DC obtained consent, obtained photographs, prepared images, reviewed reports and performed literature searches. Both DC and NC reviewed the literature and provided authorship of the text of this manuscript.

## Consent

Written informed consent was obtained from the patient for publication of this case report and accompanying images. A copy of the written consent is available for review by the Editor-in-Chief of this journal.
